# *QuickStats:* Motor-Vehicle–Traffic Death* Rates Among Persons Aged 15–24 Years and ≥25 Years — United States, 2000–2019

**DOI:** 10.15585/mmwr.mm7008a6

**Published:** 2021-02-26

**Figure:**
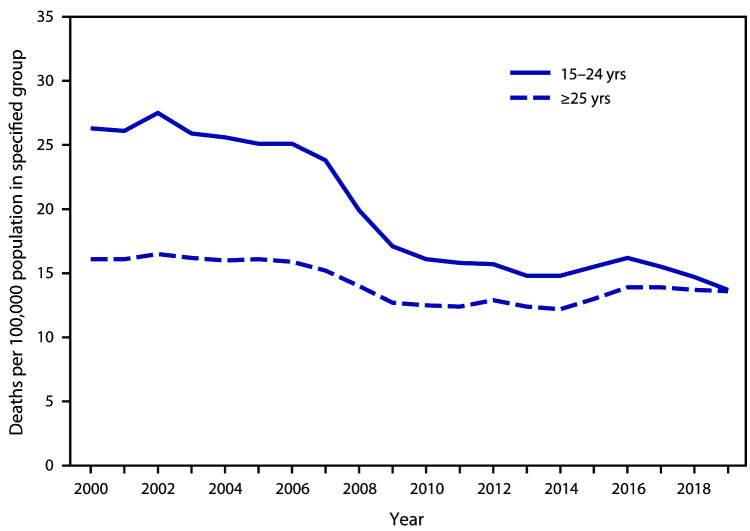
From 2000 to 2006, rates of death caused by motor-vehicle–traffic injuries among persons aged 15–24 years and ≥25 years did not change significantly. From 2006 to 2010, motor-vehicle–traffic death rates per 100,000 population declined among those aged 15–24 years, from 25.1 (2006) to 16.1 (2010), and among those aged ≥25 years, from 15.9 (2006) to 12.5 (2010). Throughout most of the period, motor-vehicle–traffic death rates were higher among persons aged 15–24 years; however, motor-vehicle–traffic death rates began to converge in more recent years, and by 2019, the difference in the rate among those aged 15–24 years (13.7) and those aged ≥25 years (13.6) was not statistically significant.

**Source:** National Center for Health Statistics, National Vital Statistics System, Mortality Data, 2000–2019. https://www.cdc.gov/nchs/nvss/deaths.htm

For more information on this topic, CDC recommends the following link: https://www.cdc.gov/transportationsafety

